# Pathologic complete response after neoadjuvant therapy for resectable esophageal squamous cell carcinoma: Endoscopic characteristics and implications

**DOI:** 10.1055/a-2625-5884

**Published:** 2025-07-23

**Authors:** Peng Yuan, Zongchao Liu, Liang Dai, Yan Yan, Yaya Wu, Keneng Chen, Wenqing Li, Qi Wu

**Affiliations:** 1 State Key Laboratory of Holistic Integrative Management of Gastrointestinal Cancers, Department of Endoscopy, Peking University Cancer Hospital & Institute, Beijing 100142, China; 2 12519 Department of Cancer Epidemiology, State Key Laboratory of Holistic Integrative Management of Gastrointestinal Cancers, Peking University Cancer Hospital & Institute, Beijing 100142, China; 3 Department of Thoracic Surgery I, Key Laboratory of Carcinogenesis and Translational Research (Ministry of Education), Peking University Cancer Hospital & Institute, Bejing, China

**Keywords:** Esophagogastroduodenoscopy, Pathologic complete response, Chemoimmunotherapy, Chemotherapy, Esophageal squamous cell carcinoma

## Abstract

**Background and study aims:**

This study aimed to identify endoscopic characteristics and develop predictive models for detecting a pathologic complete response (pCR) after neoadjuvant therapy in patients with esophageal squamous cell carcinoma (ESCC).

**Patiens and methods:**

This study enrolled 220 patients including a retrospective cohort (n = 158) and a prospective cohort (n = 62), from May 2018 to March 2023 with ESCC who received neoadjuvant chemoimmunotherapy (nCIT) or neoadjuvant chemotherapy (nCT) followed by surgery. Predictive capability of the endoscopic characteristics for pCR was developed and validated using machine learning.

**Results:**

All patients underwent endoscopic examinations before surgery but after neoadjuvant therapy. Cohort I was divided into a training set (n = 112) and an internal validation set (n = 46) at a 7:3 ratio. Seven endoscopic features were assessed: scarring; intraepithelial papillary capillary loop (IPCL) type B; depressed mucosa post-tumor disappearance; eroding mucosal changes with an uneven surface; nonsuperficial neoplastic lesions; protruded changes; and presence of cancer cells in biopsy specimens. Using these characteristics as predictors, a multivariate logistic regression model was trained to predict pCR. For further validation, data from prospective Cohorts II and III were incorporated. The model achieved 96.43% accuracy (95% confidence interval [CI] 91.11%-99.02%) in the training set, 93.48% (95% CI 82.10%-98.63%) for internal validation of Cohort I, and 96.77% (95% CI 88.83%-99.61%) in the prospective validation set.

**Conclusions:**

Endoscopic characteristics are significant predictors of pCR in patients with ESCC receiving nCIT or nCT. The predictive model demonstrated high accuracy in both derivation and validation cohorts.

## Introduction

Esophageal cancer remains a significant health burden, ranking as the seventh most common cancer in incidence and sixth in mortality worldwide 1 . It is particularly prevalent in China, accounting for over half of all annual diagnoses. Esophageal squamous cell carcinoma (ESCC) occurs predominantly in Central and Eastern Asia, representing approximately 90% of cases 1 . Standard treatment for locally advanced ESCC includes neoadjuvant chemoradiotherapy (nCRT) followed by surgery 2 ; however, the wide range of pathologic complete response (pCR) rates, ranging from 13% to 40%, suggests that some patients may undergo unnecessary surgeries post-nCRT 3 4 . The recent surge in immunotherapy, especially with immune checkpoint inhibitors (ICIs) targeting programmed cell death protein and programmed death-ligand 1, offers a novel approach to ESCC management 5 6 7 8 9 . These therapies, including neoadjuvant chemoimmunotherapy (nCIT) and chemotherapy (nCT), are poised to refine future treatment regimens as clinical trials continue to validate their effectiveness and safety 10 11 .

Precise pCR prediction is important for individualizing patient treatment strategies. This may enable some patients to avoid surgery if pCR is anticipated, thereby improving quality of life (QoL) and reducing healthcare burden. For patients experiencing a complete response, active surveillance may be a less risky option compared with traditional surgery. Nevertheless, the current challenge is to reliably distinguish between complete and incomplete responses. Conventional imaging methods, such as endoscopic ultrasound (EUS), magnetic resonance imaging (MRI), computed tomography (CT) and positron emission tomography-CT (PET-CT), are insufficient because they cannot differentiate between post-radiation changes and residual tumor tissue 12 13 14 15 . However, emerging endoscopic techniques, including narrow band imaging and blue laser imaging, represent a new frontier in pCR staging. Their enhanced capabilities for detecting subtle esophageal changes offer a compelling alternative to traditional imaging methods, thus avoiding the effects of radiation and providing a more nuanced assessment. Moreover, nCIT and nCT, compared with nCRT, may inflict less damage to the esophageal lining, thus facilitating prediction of pCR via endoscopic assessment alone.

Despite the promising potential of these endoscopic advancements, there is a notable gap in research focusing on their use for predicting pCR in ESCC following nCIT or nCT. In this study, we addressed this gap by exploring tumor-associated markers that are visible through white-light endoscopy following treatment. We identified novel endoscopic features that may be incorporated into an effective scoring system. This is not only superior to traditional imaging methods, but also shows high potential for accurately predicting pCR in patients with ESCC undergoing nCIT or nCT treatments.

## Study design and participants

### Methods

This study was conducted as a multi-cohort investigation with the primary aim of identifying endoscopic features that are predictive of a pCR in ESCC following nCIT or nCT. A pCR was stringently defined as total absence of viable tumor cells within a resected esophageal specimen with lymph nodes excluded, following standards of the Japanese Classification of Esophageal Cancer. We recruited 220 participants diagnosed with ESCC at Peking University Cancer Hospital who were candidates for esophagectomy following neoadjuvant therapy. Inclusion criteria were individuals undergoing nCT or nCIT, who had endoscopic examinations, whereas those who had preoperative therapy for stage IV disease or had unresectable tumors were excluded from the study. All participants underwent curative Mckeown esophagectomy. A standard two-field lymph node dissection was performed on all patients, and if cervical lymph node metastasis was suspected, a three-field lymph node dissection was performed. Criteria for radical R0 resection were based on intraoperative gross judgment and postoperative pathological confirmation of a negative surgical margin.

Participants were sequentially recruited from three independent cohorts, designated as Cohorts 1 (n = 158), 2 (n = 23), and 3 (n = 39). Cohort 1 was the basis of our retrospective analysis and identification of key endoscopic characteristics predictive of pCR, which included 158 subjects enrolled from May 2018 to August 2022. Cohorts 2 and 3, consisting of 23 and 39 participants, respectively, were recruited for prospective validation between August 2022 and March 2023. Patients in these two cohorts underwent gastroscopy and results were interpreted by different endoscopic physicians. Machine learning models were developed for pCR prediction, which were internally validated using data from Cohort 1 and subsequently validated with independent data from Cohorts 2 and 3. Additional data including age, tumor location, tumor stage, number of neoadjuvant therapy cycles, and different ICIs were collected for all cohorts to enhance robustness of findings. The study protocol was approved by the Institutional Review Board (IRB_ of Peking University Cancer Hospital with the IRB approval number being (2021KT14) and informed consent was obtained from all participants before surgery.

### Neoadjuvant therapy

According to the study design, subjects underwent two to four cycles of neoadjuvant chemotherapy or chemoimmunotherapy before surgery over a 21-day cycle period. The regimen typically included cisplatin or carboplatin with paclitaxel or albumin-bound paclitaxel, whereas chemoimmunotherapy included these drugs combined with immunotherapy, such as pembrolizumab, sintilimab, toripalimab, tislelizumab, or camrelizumab. Following neoadjuvant treatment, participants underwent McKeown esophagectomy to achieve a radical R0 resection, which is defined by no tumor cells at the surgical margins. This surgery involved meticulous lymph node dissection consistent with the JES (Japan Esophageal Society) classification 16 and targeting removal of at least 20 nodes. The extent of lymphadenectomy was determined by the anatomical location of the tumor, comprehensively encompassing the upper, middle, and lower mediastinal nodes, as well as abdominal lymph nodes, to thoroughly address lymphatic drainage specific to thoracic esophageal cancer.

### Endoscopic characteristics

**Fig. 1:**
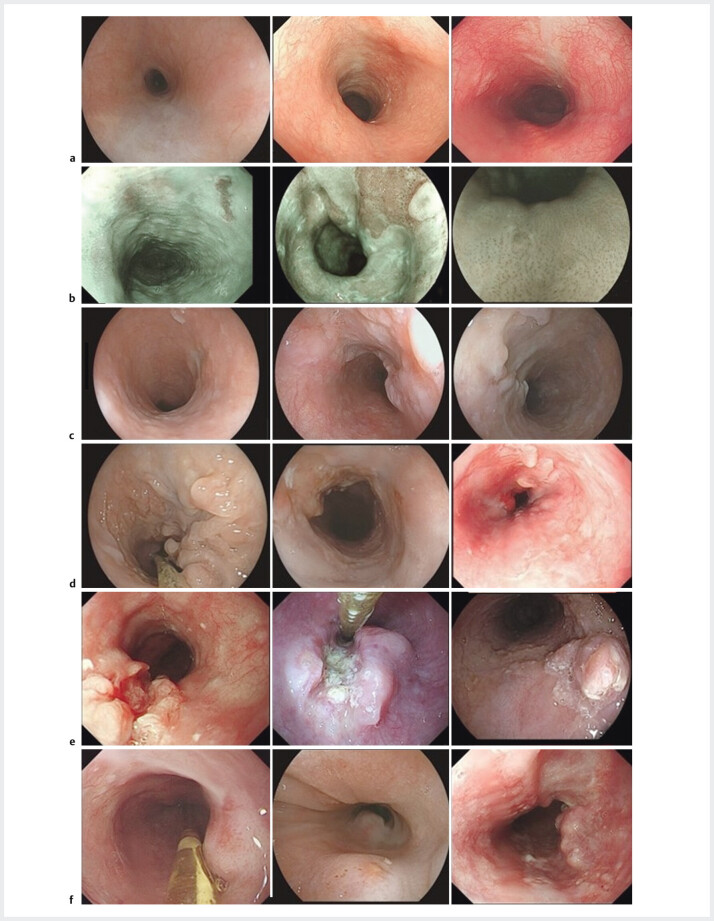
Endoscopic characteristics. **a** Scarring. **b** IPCL type B. **c** Depressed mucosa after tumor disappearance. **d** Eroding mucosal changes with an uneven surface. **e** Nonsuperficial neoplastic lesions. **f** Protruded changes.

We performed endoscopic evaluations for all participants approximately 4 weeks after completing their neoadjuvant therapy and within 1 week prior to surgery to assess treatment response. Endoscopic assessment strategy was meticulously designed by two independent gastrointestinal endoscopists (YP and YY), with WQ providing resolution in cases of disagreement. Identification of specific endoscopic characteristics was based on a detailed review of macroscopic tumor changes following nCIT or nCT. We used a systematic approach to identify features most predictive of pathological outcomes, which was independent of pathological knowledge to maintain assessment impartiality. Based on this approach, we established seven key endoscopic characteristics: 1) scarring; 2) intraepithelial papillary capillary loop (IPCL) type B represents irregular, tortuous, and dilated capillary loops detected, indicative of tumor vasculature; 3) depressed mucosa post-tumor disappearance; 4) eroding mucosal changes with an uneven surface; 5) nonsuperficial neoplastic lesions; 6) protruding changes ( Fig. 1 ); and 7) presence of cancer cells in biopsy specimens. Characteristics one through six were documented in a binary format, either present or absent, whereas characteristic seven was uniquely classified into three categories: “not captured,” indicating technical challenges that precluded obtaining adequate samples; “detected,” indicating presence of cancer cells; and “not detected,” indicating absence of cancer cells. This classification reflects variability in biopsy outcomes during endoscopic assessment. Selection of these characteristics emerged from an iterative process that included a comprehensive review of endoscopic images, pathological findings, and expert consensus. To delineate prevalence and correlation of these characteristics with pathological outcomes, we visualized their distributions across the cohorts, which enabled us to refine their predictive value for pCR.

### Statistical analysis

The primary outcome was manifestation of pCR. We used machine learning pipelines to elucidate predictive capability of candidate endoscopic characteristics associated with pCR in patients with ESCC treated with nCT or nCIT. We randomly divided Cohort 1 into a training set (n = 112) and an internal validation set (n = 46) at a 7:3 ratio. Using seven endoscopic characteristics as predictors, a multivariate logistic regression model was trained to predict pCR. The optimal model was selected through 10 iterations repeated with five fold cross-validation based on the training set and subsequent validation with the internal validation set. Model efficacy was subsequently determined using the internal validation set. For further prospective validation, data from the prospective Cohorts 2 (n = 23) and 3 (n = 39) were incorporated. To assess the influence of biopsy on model discrimination, we repeated the entire multivariable logistic-regression pipeline after removing biopsy information and compared model performance between the original seven-feature model and the resulting six-feature model.

Model performance was evaluated by calculating sensitivity, specificity, positive predictive value (PPV), negative predictive value (NPV), and area under the receiver operating characteristic curve (AUC). To enhance clinical utility, we developed a scoring system based on a nomogram. The scoring system was built using a linear combination of the logistic regression model coefficients and identified endoscopic characteristics, allowing to quantify probability of achieving a pathologic complete response (pCR) for each individual. To determine utility of the scoring system, we examined the association of tumor stage following neoadjuvant therapy (ypT) and lymph node status (ypN) with individual-level scores for pCR prediction either separately by cohorts or combining them using the Jonckheere-Terpstra trend test. A two-sided *P* value of 0.05 was considered statistically significant.

## Results

### Baseline clinical characteristics of patients

Table 1 shows baseline characteristics of the three cohorts. Among all cohorts, participants ranged in age from 39 to 85 years, and they were predominantly male, with 80.4% in Cohort 1, 95.7% in Cohort 2, and 89.7% in Cohort 3. The lower third of the esophagus was the most frequent cancer location, which was observed in 50% of Cohort 1 and 56.5% of Cohort 2. A significant proportion of patients were initially diagnosed at clinical stage cT3. Median number of nCIT cycles was two. For the nCIT group, the most commonly used ICI was tislelizumab, which accounted for 33.7%, 61.9%, and 30% in Cohorts 1, 2, and 3, respectively. With respect to treatment outcomes, 32 patients (20.3%) in Cohort 1 achieved a pT0 stage, indicating no detectable tumor cells post-treatment, whereas six (26.1%) and seven patients (17.9%) in Cohorts 2 and 3, respectively, achieved pT0 stage. Remarkably, the preferred presurgical treatment for 91.3% of the Cohort 2 patients was chemoimmunotherapy. Median time from endoscopy to esophagectomy was 6 days for Cohort 1, 2 days for Cohort 2, and 5 days for Cohort 3.

**Table:** **Table 1** Baseline clinical characteristics of the included patients.

	Cohort I	Cohort II	Cohort III
	**n = 158**	**n = 23**	**n = 39**
**Age**	63 (39–81)	65 (47–85)	63 (46–76)
**Male (%)**	127 (80.4)	22 (95.7)	35 (89.7)
**Tumor location (%)**			
Upper third	26 (16.5)	0	9 (23.0)
Middle third	53 (33.5)	10 (43.5)	18 (46.2)
Lower third	79 (50.0)	13 (56.5)	12 (30.8)
**Tumor length (%)**			
< 5 cm	92 (58.2)	6 (26.1)	21 (53.8)
≥ 5 cm	66 (41.8)	17 (73.9)	18 (46.2)
**Clinical T stage (%)**			
cT1	5 (3.2)	0	0
cT2	22 (13.9)	4 (17.4)	8 (20.5)
cT3	117 (74.1)	17 (73.9)	30 (76.9)
cT4	14 (8.9)	2 (8.7)	1 (2.6)
**Clinical N stage (%)**			
cN0	13 (8.2)	1 (4.3)	4 (10.3)
cN1	66 (41.8)	7 (30.4)	13 (33.3)
cN2	68 (43.0)	11 (47.8)	19 (48.7)
cN3	11 (7.0)	4 (17.4)	3 (7.7)
**Neoadjuvant therapy regimen (%)**			
nCIT	83 (52.5)	21 (91.3)	30 (76.9)
nCT	75 (47.5)	2 (8.7)	9 (23.1)
**No. neoadjuvant therapy cycles (%)**			
nCIT	2 (1–7)	2 (2–6)	2 (2–4)
nCT	2 (1–7)	2.5 (2–3)	2 (1–4)
**ICIs (%)**			
Pembrolizumab	14 (16.9)	1 (4.8)	6 (20.0)
Sintilimab	7 (8.4)	0	2 (6.7)
Toripalimab	15 (18.1)	4 (19.0)	7 (23.3)
Camrelizumab	14 (16.9)	3 (14.3)	6 (20.0)
Tislelizumab	28 (33.7)	13 (61.9)	9 (30.0)
Others	5 (6.0)	0	0
**Pathologic T stage (%)**			
pT0	32 (20.3)	6 (26.1)	7 (17.9)
pTis	7 (4.4)	0	3 (7.7)
pT1a	8 (5.1)	4 (17.4)	2 (5.2)
pT1b	24 (15.2)	3 (13)	10 (25.6)
pT2	28 (17.7)	4 (17.4)	5 (12.8)
pT3	57 (36.1)	6 (26.1)	12 (30.8)
pT4	2 (1.3)	0	0
**Pathologic N stage (%)**			
pN0	89 (56.3)	12 (52.2)	26 (66.7)
pN1	41 (25.9)	6 (26.1)	8 (20.5)
pN2	22 (13.9)	4 (17.4)	5 (12.8)
pN3	6 (3.8)	1 (4.3)	0
**Time intervals (days)**			
End of nCT/nCIT until esophagectomy	37 (20–223)	41 (26–67)	42 (22–136)
End of nCT/nCIT until endoscopy	27 (0–200)	39 (23–53)	30 (0–133)
End of endoscopy until esophagectomy	6 (0–114)	2 (0–14)	5 (0–100)
ICI, immune checkpoint inhibitor; nCIT, chemoimmunotherapy; nCT, chemotherapy.			

### Evaluation of endoscopic characteristics for predicting pathological complete response

**Fig. 2:**
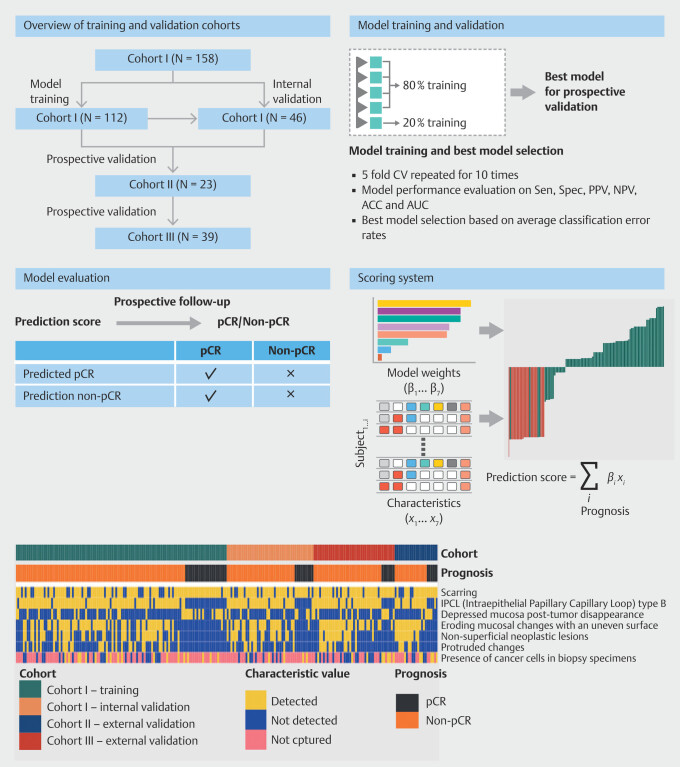
Study workflow. A total of 220 subjects from three independent cohorts were sequentially recruited. Cohort 1, consisting of 158 subjects who were enrolled from May 2018 to August 2022, served as the foundation for the retrospective analysis and identification of key endoscopic characteristics predictive of pathologic complete response (pCR). Cohorts 2 and 3, recruited for prospective validation from August 2022 to March 2023, consisted of 23 and 39 participants, respectively. Seven key endoscopic characteristics were identified and incorporated to develop machine learning models for pCR prediction. The models were primarily trained and internally validated based on data from Cohort I, and prospectively validated using data from the independent Cohorts 2 and 3. The distribution of the seven key endoscopic characteristics and pCR/non-pCR status across the three cohorts is shown by heatmaps. CV, cross-validation; ACC, accuracy; AUC, area under the curve; NPV, negative predictive value; PPV, positive predictive value; Sen, sensitivity; Spec, specificity; p-CR, pathologic complete response; Non-pCR, non-pathologic complete response.

**Fig. 3:**
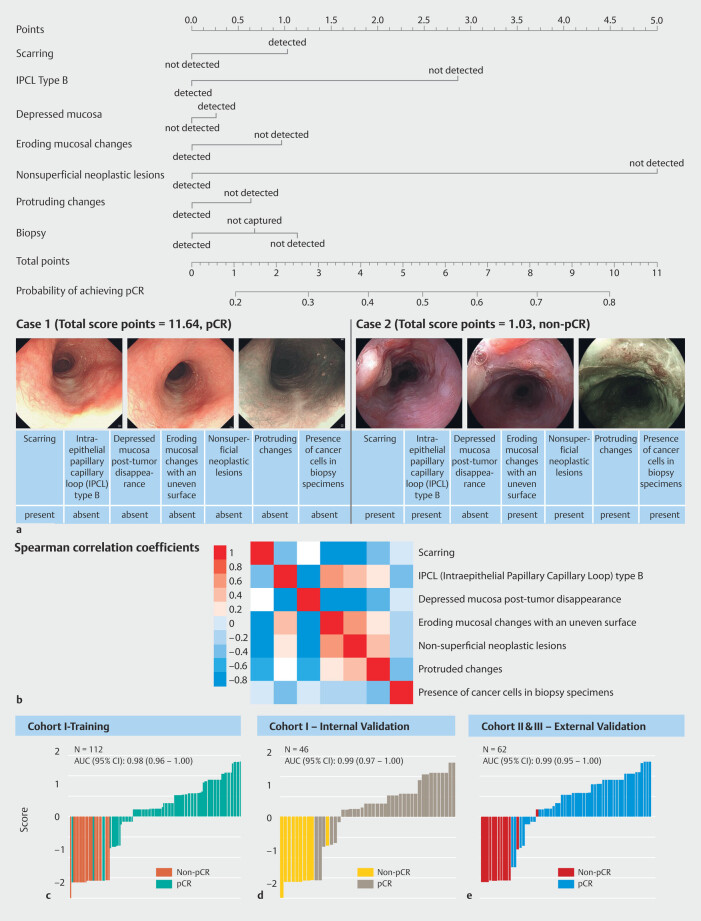
Endoscopic characteristic analysis and predictive performance of pCR. **a** Nomogram for predicting pathologic complete response (pCR) based on endoscopic characteristics. Each endoscopic feature is assigned a score along the “Points” axis based on its presence or absence. The total score is determined by summing the individual scores and locating this sum along the “Total Points” axis. The corresponding point on the “Probability of achieving pCR” axis indicates the estimated probability of pCR. The nomogram also highlights the relative importance of each characteristic in predicting pCR. On the right side of the nomogram, two typical cases are presented, showing the presence of each characteristic, their respective scores, and the final pCR or non-pCR outcomes. **b** Pairwise correlations among seven key endoscopic characteristics were demonstrated, revealing a moderately positive correlation (Spearman’s r = 0.67, *P <* 0.05) between the characteristics of eroding mucosal changes with an uneven surface and nonsuperficial neoplastic lesions, whereas weaker correlations were observed for the others (Spearman’s r < 0.5, *P* < 0.05). The heatmap cells reflect these correlations, with color gradations ranging from blue (low correlation) to red (high correlation). **c** , **d** , **e** Scoring system efficacy for predicting a pCR. Individual pCR likelihood scores, based on integration of the seven key characteristics, were computed for subjects across Cohorts 1, 2, and 3. Each bar in the chart represents an individual’s score, color-coded to indicate pCR status. For each cohort, the area under the receiver operating characteristic curve (AUC) and the 95% CI were determined through 2000 stratified bootstrap replicates.

Distribution of seven established endoscopic characteristics was assessed for the three cohorts ( Fig. 2 ). The nomogram illustrating the predictive factors for pCR is presented in Fig. 3 **a** . A moderately positive correlation (Spearman’s r = 0.67, *P* < 0.05) was observed between the characteristics of eroding mucosal changes with an uneven surface and non-superficial neoplastic lesions, whereas the other characteristics exhibited weak correlations (Spearman’s r < 0.5, *P <* 0.05) ( Fig. 3 **b** ). The model demonstrated consistent performance, with an accuracy of 96.43% (95% confidence interval [CI] 91.11%-99.02%) in the training set and 93.48% (95% CI 82.10%-98.63%) for the internal validation using Cohort 1. Cohorts 2 and 3 were aggregated as the prospective validation set with an accuracy of 96.77% (95% CI 88.83%–99.61%), as listed in Table 2 . To facilitate clinical application, a scoring system was established using a nomogram based on the model weights ( Fig. 3 **a** ), which integrated individual-level scores indicating likelihood of a pCR. For each individual, the score was computed based on the criteria: Score = 1.03 × *I* (Scarring - detected) + 2.86 × *I* (IPCL type B - not detected) + 0.27 × *I* (Depressed mucosa after tumor disappearance - detected) + 0.97 × *I* (Erodiing mucosal changes with an uneven surface - not detected) + 5.00 × *I* (Non-superficial neoplastic lesions - not detected) + 0.64 × *I* (Protruded changes - not detected) + 1.13 × *I* (Presence of cancer cells in biopsy specimens - not detected) + 0.68 × *I* (Presence of cancer cells in biopsy specimen - not captured), where *I* ( ) is an indicator function that denotes presence or absence of a specified endoscopic characteristic. The scores showed a high predictive performance with an AUC of 0.98 (95% CI 0.96–1.00) in the training set, 0.99 (95% CI 0.97–1.00) in the internal validation set, and 0.99 (95% CI 0.95–1.00) in the prospective validation set ( Fig. 3 **c** , Fig. 3 **d** , Fig. 3 **e** ). To verify that model discrimination did not depend solely on the biopsy variable, we refit the multivariable logistic model using only the six macroscopic endoscopic features and compared its performance with the original seven-feature model. As shown in Table 2 , the six-feature visual model exhibited nearly identical sensitivity, specificity, NPV, PPV, accuracy, and AUC across the training, internal validation, and prospective validation cohorts ( Table 2 ). These results confirm that macroscopic endoscopic findings alone retain strong predictive power for pCR. The ideal cut-off value for predicting a pCR was determined by aggregating data from all three cohorts. This analysis indicated that a cut-off score of −0.784 after standardization, which corresponds to an AUC of 0.98, optimally distinguished the outcomes.

**Table:** **Table 2** Model performance in predicting pCR across cohorts 1, 2, and 3.

Cohort / Metric	7-feature model (with biopsy)	6-feature model (no biopsy)
Training (Cohort 1, n = 112)		
Sen	0.9556	0.9556
Spec	1	1
NPV	0.8462	0.8462
PPV	1	1
ACC (95% CI)	0.9643 (0.9111~0.9902)	0.9643 (0.9111~0.9902)
AUC (95% CI)	0. 9831 (0. 9628~1.0000)	0. 9826 (0. 9629~1.0000)
Cohort I - prospective validation (n = 46)		
Sen	0.9444	0.9444
Spec	0.9	0.9
NPV	0.8182	0.8182
PPV	0.9714	0.9714
ACC (95% CI)	0.9348 (0.8210~0.9863)	0.9348 (0.8210~0.9863)
AUC (95% CI)	0.9889 (0.9681~1.0000)	0.9833 (0.9577~1.0000)
Cohorts 2 and 3 - prospective validation (n = 62)		
Sen	1	1
Spec	0.8462	0.8462
NPV	1	1
PPV	0.9608	0.9608
ACC (95% CI)	0.9677 (0.8883~0.9961)	0.9677 (0.8883~0.9961)
AUC (95% CI)	0.9553 (0.8737~1.0000)	0.9608 (0.8737~1.0000)
The 7-feature model includes all seven features (scarring, IPCL type B, depressed mucosa, eroding changes, nonsuperficial lesions, protrusions, and biopsy results) in preoperative endoscopy, whereas the 6-feature model omits the biopsy evaluation. ACC, accuracy; AUC, area under the curve; CI, confidence interval; NPV, negative predictive value; PPV, positive predictive value; Sen, sensitivity; Spec, specificity.		

We tested associations of ypT/ypN with individual-level scores for pCR prediction by aggregating participants from all cohorts. The scores revealed a significant decreasing trend concomitant with tumor staging for either ypT stages ( *P* < 0.001) or ypN stages ( *P* = 0.03) ( Table 3 ).

**Table:** **Table 3** Association between tumor staging and integrated scores from endoscopic characteristics in ypT and ypN categories.

Stage	N	Score (mean ± SD) ^*^	Score range	*P* ^†^
ypT				
0	45	6.14 ± 3.41	(0.27–10.40)	< 0.001
tis	10	5.77 ± 3.28	(0.27–10.40)	
1a	14	5.37 ± 3.25	(1.23–10.14)	
1b	37	5.30 ± 3.18	(0.27–10.40)	
2	37	5.10 ± 3.53	(0.90–10.40)	
3	75	4.99 ± 3.71	(0.27–10.40)	
4	2	3.40 ± 3.54	(0.90–5.90)	
ypN				
0	127	5.70 ± 3.29	(20.16–174.55)	0.03
1	55	5.60 ± 3.46	(20.16–173.77)	
2	31	5.51 ± 3.73	(36.92–173.77)	
3	7	4.82 ± 2.60	(79.65–151.89)	
^*^ Scores are presented as mean ± standard deviation (SD). ^†^ The Jonckheere-Terpstra trend test was used to analyze the trend across stages in both ypT and ypN categories. pN, lymph node status; ypT, tumor stage following neoadjuvant therapy.				

## Discussion

Research into esophageal cancer treatment has highlighted the challenge of accurately predicting pCR following neoadjuvant therapy. Traditional assessment techniques, including endoscopic evaluation and imaging, such as PET-CT and diffusion weighted-MRI, have demonstrated limited efficacy in predicting pCR 17 18 . Of note, there have been limited studies focused on predicting pCR after nCT or nCIT. We addressed this gap by examining seven endoscopic signs indicative of pCR in ESCC patients post-nCT or nCIT. Drawing data from three separate cohorts and using machine learning for analysis, we identified post-treatment endoscopic features as markers for pCR, resulting in development of a predictive scoring system. This offers a nuanced approach to ESCC management, emphasizing personalized treatment and efficient use of medical resources.

Our findings contribute to management of locally advanced esophageal cancer, particularly with respect to emerging neoadjuvant therapies. Neoadjuvant therapy has significant potential in downstaging of disease and enabling a subset of patients to achieve a clinical complete response (cCR). This is consistent with our observed endoscopic characteristics that are predictive of pCR. Ethical implications of high remission rates, juxtaposed with risks of esophagectomy, underscore the need for a paradigm shift toward individualized treatment strategies, such as active monitoring, which our study advocates.

Based on endoscopic characteristics that we selected, our prediction model revealed sensitivity, specificity, PPV, and NPV of 95.56%, 100%, 100%, and 84.62%, respectively, for predicting pCR. In the context of nCIT or nCT, these metrics surpass those described in previous studies. This result is quite different from the prevailing sentiment that endoscopic evaluations are ineffective at predicting pCR following nCRT 18 19 . Recently, the comprehensive review by Van der Wilk et al. 3 has shown promising survival rates and comparable mortality risks between patients undergoing active monitoring and those receiving surgery. These studies resonate with the ability of our model to identify patients who might benefit from less invasive management post-neoadjuvant therapy. Conversely, the active monitoring approach, which could reserve esophagectomy for patients with confirmed or suspected residual disease, is being rigorously tested in randomized trials, such as the SANO trial 20 21 . Our predictive scoring system may also improve the selection process for stratifying patients, ensuring that those with a high likelihood of a pCR could avoid surgery. Therefore, our model adds an important layer to our understanding of how endoscopic indicators post-neoadjuvant therapy can inform clinical decisions, thus striking a balance between treatment efficacy and patient QoL. Integrating endoscopic characteristics with advanced imaging can refine pCR assessments 22 and further studies are needed to optimize therapeutic response evaluations for nCIT- and nCT-treated ESCC patients.

Among endoscopic characteristics detailed in this study, scarring, IPCL type B, and biopsy were associated with postoperative pCR. Previous studies have identified scarring as a potential marker of pCR 16 23 , which is consistent with our findings. IPCL is a histological characteristic observed during the early stages of esophageal cancer 24 25 . In 2011, the Japanese Esophageal Cancer Association introduced a classification system for IPCL, which divided it into types A and B. IPCL type B is routinely used to determine invasion depth of early esophageal cancer. In the present study, we observed that manifestation of IPCL type B vessels often signals residual tumor. Tumors with macroscopically diagnosed invasion confined to the submucosa are categorized as superficial, which suggests that nonsuperficial lesions denote potential residual tumor. Importantly, although available biopsy results add tissue-based information to the prediction model, they were incorporated as a procedural adjunct rather than as an independent endoscopic feature. Within the multivariable framework, biopsy status serves to enrich but not override endoscopic assessment. Therefore, interpretation of the scoring system should be based on the combined pattern of all endoscopic findings rather than biopsy status in isolation. This holistic design ensures that any single false-negative or false-positive biopsy could be counterbalanced by the remaining endoscopic features. The slightly depressed mucosal pattern observed endoscopically after tumor disappearance resembles a stamped mark, similar to lunar craters. Pathological analysis often reveals fibrosis, mononuclear lymphocyte infiltration, multinucleated giant cell responses, and chronic inflammation. Of note, patients with scant residual tumor cells often exhibit IPCL type B within these post-neoadjuvant chemotherapy or immunotherapy craters, which supports our observations. Endoscopic characteristics, such as eroding and irregular mucosal surfaces or protrusions, highlighted in the Japanese guidelines for assessing response to neoadjuvant chemoradiotherapy (nCRT) in esophageal cancer 16 , suggest submucosal invasion, which accounts for high false-negative rates in biopsies. To reduce these errors, “bite on bite” biopsies and fine-needle aspirations are recommended 22 ; however, false-negative rates for detecting residual cancer still range from 1% to 10%, necessitating careful implementation of these approaches considering risk of esophageal perforation.

In the context of organ preservation strategies, rigorous assessment of patients who appear to achieve a cCR is important. Accurate evaluation of a cCR is challenging because current methods, such as CT, MRI, and EUS, are insufficient at identifying nuances of post-therapeutic tissue changes, such as scarring, and can miss up to 10% of residual tumors 26 27 28 . Our model, which seeks to predict pCR, indirectly contributes to this dilemma. Although pCR is ultimately confirmed by pathological examination post-surgery, predicting it accurately can identify patients who are likely to have a true cCR. This enables informed decisions to be made regarding patients who are suitable for nonsurgical management, such as active monitoring, versus those who may benefit from surgery. Thus, while we do not directly measure cCR, our predictive model provides a valuable tool for the preoperative phase to predict likelihood of pCR and, by extension, true cCR.

The observed significant decreasing trend in scores across increasing ypN stages highlights the relationship between lymph node metastasis and endoscopic features predictive of pathological complete response (pCR). Elevated ypN stages are often associated with more unfavorable endoscopic characteristics, such as IPCL type B and non-superficial neoplastic lesions, both of which contribute to lower total scores. Our findings support the robustness of the scoring system, demonstrating its capacity to capture not only the pathological burden at the primary tumor site but also at regional lymph nodes. Future research should explore integration of specific biomarkers or advanced imaging techniques to further enhance predictive power of the scoring system for lymph node metastasis.

This study has some limitations. First, although two of the three cohorts were prospective, lending a degree of credibility and robustness to our findings, the sample size was relatively small, so the study may not sufficiently represent the broader population. In addition, although Cohorts 2 and 3 were evaluated by different physicians, all evaluations and treatments were conducted within the same institution, limiting the strict definition of external validation across multiple institutions. However, use of independent endoscopists might help reduce bias associated with physician interpretation. Second, the study may fail to identify diminutive or concealed residual tumor cells situated in the submucosa, potentially inflating pCR rates; however, we attempted to mitigate this concern by incorporating endoscopic characteristics suggestive of submucosal invasion into the prediction model. Third, there is some subjectivity inherent in interpreting endoscopic observations; however, endoscopists validating our prediction model both achieved comparable accuracy in pCR prediction. Future studies should include a larger cohort and involve multiple institutions to enhance the model validation.

## Conclusions

In summary, our study highlights the importance of specific endoscopic characteristics in predicting pCR in patients with ESCC undergoing nCIT or nCT. To refine assessment of treatment response for ESCC, it is essential to fully define the role of endoscopy in pCR evaluation. Integrating these findings with other diagnostic methods, such as imaging and blood biomarkers, may provide enhanced accuracy in predicting pCR and stratifying patients for treatment.
